# Structural basis for antibiotic resistance by chloramphenicol acetyltransferase type A in *Staphylococcus aureus*

**DOI:** 10.1038/s41598-025-18365-4

**Published:** 2025-10-23

**Authors:** Kaiyue Wang, Junwei Chen, Wanfang Li, Min Wang, Tingwen Fan, Dongbo Bu, Sheng Ye

**Affiliations:** 1 School of Biomedical Science and Medicine Engineering, Beihang University, Beijing, 100191 China; 2 Key Laboratory of Big Data-based Precision Medicine (Beihang University), Ministry of Industry and Information Technology, Beijing, 100862 China; 3 School of Arts and Sciences, Rutgers University, New Brunswick, NJ 08901 USA; 4 State Key Laboratory of Biomacromolecules, Institute of Biophysics, Chinese Academy of Sciences, Beijing, 100101 China; 5 State Key Laboratory of Microbial Diversity and Innovative Utilization, Institute of Microbiology, Chinese Academy of Sciences, Beijing, 100101 China; 6 SKLP, Institute of Computing Technology, Chinese Academy of Sciences, Beijing, 100190 China; 7 University of Chinese Academy of Sciences, Beijing, 100080 China; 8 School of Engineering Medicine, Beihang University, Beijing, 100191 China

**Keywords:** *Staphylococcus aureus*, Antibiotic resistance, Chloramphenicol acetyltransferase, Chloramphenicol, Fusidic acid, Structural biology, X-ray crystallography

## Abstract

Methicillin-resistant *Staphylococcus aureus* (MRSA) exemplifies high-level antibiotic resistance in this major human pathogen. Its resistance to chloramphenicol is majorly conferred by enzymatic inactivation via chloramphenicol acetyltransferases (CATs). This modification sterically blocks the antibiotic’s ribosomal binding and thus neutralizes its inhibitory potency. Although CATs have been structurally studied across diverse bacteria species, the structures of *S. aureus* CATs (saCATs) have remained uncharacterized. To address this gap and elucidate species-specific resistance mechanisms, we determined the first high-resolution crystal structure of saCAT1, the prototypical saCAT enzyme. Structural analysis delineates the active site architecture and reveals the molecular basis for substrate recognition of both chloramphenicol and fusidic acid (FA). Further enzymatic assays demonstrated that the *K*_m_ value against chloramphenicol is 16.9 µM, and the *K*_i_ value of the inhibitor FA is 83.7 µM, indicating that the inhibitory capacity of FA is relatively limited. These findings provide an essential structural framework for understanding chloramphenicol resistance in *S. aureus* and facilitate the rational design of novel antimicrobial strategies to combat multidrug-resistant pathogens.

**Supplementary Information:**

The online version contains supplementary material available at 10.1038/s41598-025-18365-4.

## Introduction

*Staphylococcus aureus* is a pathogenic bacterium widely colonizing human skin and mucosal surfaces, capable of causing a broad spectrum of clinical infections, including skin abscesses, pneumonia, and sepsis^1^. As a major causative agent of both healthcare-associated and community-acquired infections, *S. aureus* has exhibited high-level antibiotic resistance in recent years, which is primarily driven by antimicrobial misuse^2,3^. The selective pressure has led *S. aureus* to develop or acquire multiple resistance genes. Some strains have evolved into so-called “superbugs”—pathogens resistant to multiple classes of antimicrobial agents^4^. In particular, infections caused by methicillin-resistant *S. aureus* (MRSA) are associated with increased mortality and pose a serious threat to global public health^5,6^. The rise of antimicrobial resistance (AMR) has escalated into a global health crisis, with AMR-related deaths surpassing those from HIV/AIDS or malaria^7^. This alarming trend highlights the critical need to unravel resistance pathways and accelerate mechanism-based therapeutics^8^.

**Fig. 1:**
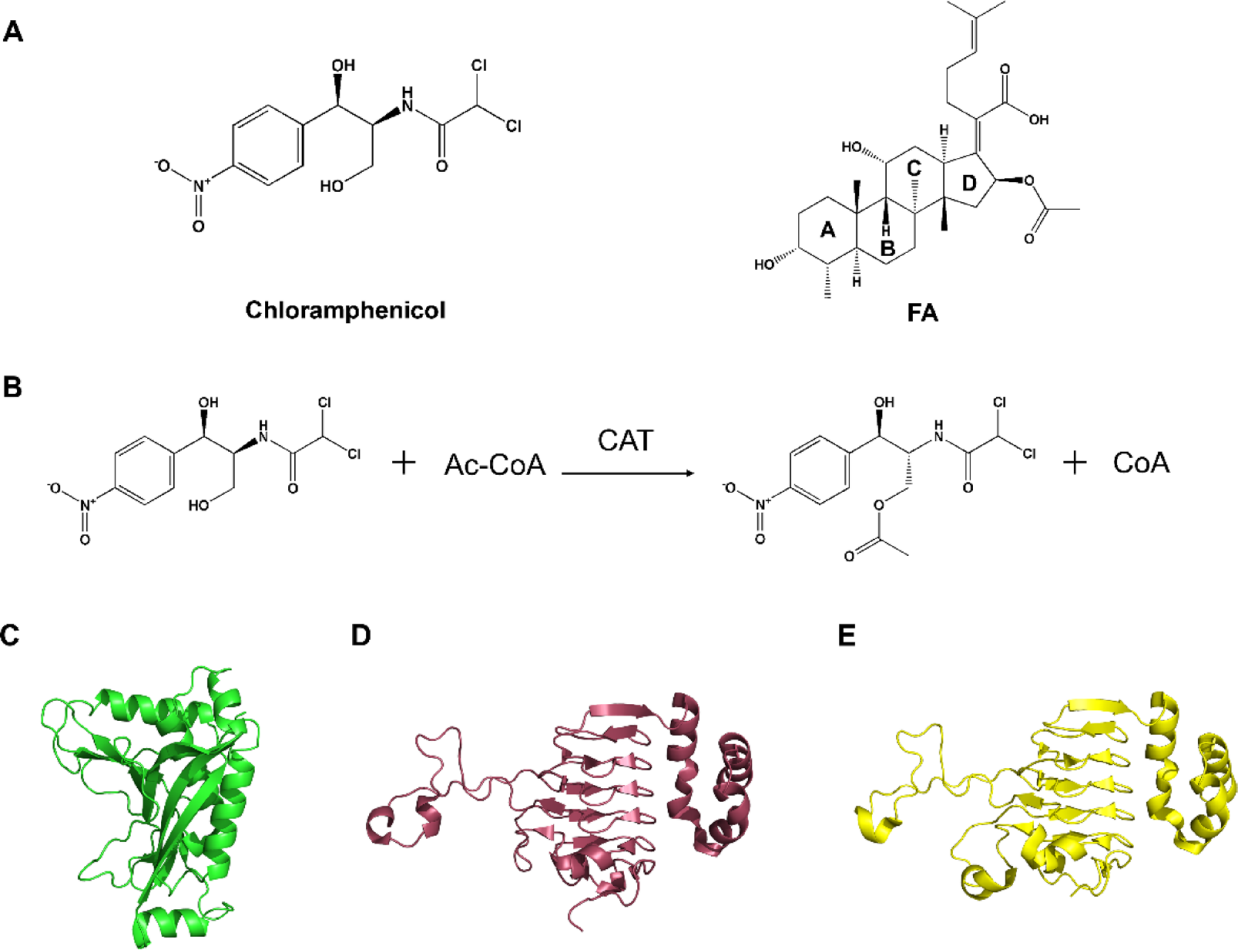
CAT-catalyzed reaction and representative structures of different CAT types. (**A**) Chemical structure of chloramphenicol (left) and FA (right). (**B**) CATs catalyze the O-acetyl transfer of an acetyl group from acetyl coenzyme A (Ac-CoA) to the 3′-hydroxyl group of chloramphenicol. (**C**) Type-A CAT from *E. coli* (PDB ID: 3CLA^21^ in green. (**D**) Type-B CAT from *Vibrio cholerae* (PDB ID: 6PUA^22^ in magenta. (**E**) Type-C CAT from *Aliivibrio fischeri* (PDB ID: 5UX9) in yellow.

To combat *S. aureus* infections, chloramphenicol (Fig. 1A) was once widely used in clinical treatment^9^. Chloramphenicol exerts its antibacterial effect by specifically binding to the bacterial 50 S ribosomal subunit and blocking peptide chain elongation^10,11^. Although its clinical application has been limited due to potential adverse effects, chloramphenicol remains an important therapeutic option in some drug-resistant infections, particularly in resource-poor health systems, because of its broad-spectrum activity, high efficacy, and low cost^12,13^. However, the prolonged use of this antibiotic has led to the evolution of resistance mechanisms in *S. aureus* and other bacterial species. The most common resistance mechanism involves enzymatic inactivation via the expression of chloramphenicol acetyltransferase (CAT)^11^. This enzyme catalyzes the transfer of an acetyl group (CH₃CO–) from acetyl-CoA to the hydroxyl group (–OH) of chloramphenicol, resulting in the formation of acetylated chloramphenicol (Fig. 1B). The modified chloramphenicol derivative demonstrates significantly reduced binding affinity for the 50 S ribosomal subunit, thereby neutralizing its inhibitory activity against bacterial protein synthesis^11,14,15^.

Chloramphenicol acetyltransferases (CATs) historically bifurcate into two evolutionarily distinct classes: type-A (16 subgroups) and type-B (5 subgroups), with intra-subgroup sequence identities exceeding 80%^10,11^. A prototypical type-A CAT is CATIII, encoded on the R387 plasmid of *Shigella flexneri*^16^ whereas the inaugural type-B representative CATB1 originates chromosomally in *Agrobacterium tumefaciens*^17^. These two classes share merely ~ 12% sequence similarity, exhibiting profound molecular divergence. Recently, Zhang et al. identified a phylogenetically divergent third class, type-C CATs, encoded in *Vibrio parahaemolyticus* and its closely related species^18^. Naturally occurring type-C CAT variants demonstrate heterogeneous chloramphenicol resistance profiles in *Escherichia coli*. Critically, comparative enzymology reveals type-A CATs possess superior chloramphenicol affinity relative to both type-B and type-C enzymes^19^. Furthermore, type-A CATs uniquely exhibit fusidic acid (FA, Fig. 1A) binding competency –– a functional signature absent in type-B and type-C isoforms^20^. Despite functioning universally as homotrimers, CAT enzymes manifest two fundamentally distinct architectural blueprints. Type-A CATs exhibit a canonical α/β fold with a three-domain configuration, which has been described as a open-faced sandwich (Fig. 1C)^21^. Conversely, type-B and type-C CATs share a conserved hexapeptide repeat fold as their structural scaffold, exhibiting pronounced mutual similarity in core architecture (Fig. 1D and E)^22^. This tripartite structural divergence correlates with the enzymes’ differential antibiotic recognition profiles, wherein the expanded binding geometry of type-A CATs accommodates both chloramphenicol and FA––a capability absent in the more compact type-B/C active sites. The difference indicates distinct catalytic trajectories in CAT’s antibiotic resistance evolution.

Despite extensive structural studies on CATs across bacterial species^15,19,22–25^, the structural characterization of CATs derived from *S. aureus* (saCATs) remain insufficiently elucidated, hindering a comprehensive understanding of its role in antimicrobial resistance. The saCAT family comprises four manually curated members (saCAT1-4), with saCAT1 serving as the prototypical enzyme. In this study, we solved the high-resolution crystal structure of saCAT1, enabling systematic analysis of its active site topology and mapping molecular interaction mechanisms with substrate chloramphenicol and fusidic acid through computational docking. Further comparative structural analyses reveal saCAT1’s unique evolutionary adaptations in resistance determinant organization, substrate recognition geometry, and catalytic residue configuration. In parallel, we assessed the chloramphenicol acetyltransferase activity of saCAT1 and evaluated its inhibition by FA. As the first three-dimensional structure in the saCAT family, this study establishes a structural foundation for rational design of next-generation inhibitors targeting multidrug-resistant *Staphylococcal* strains.

## Result

### Sequence homology indicates that saCAT1 belongs to type-A CAT family

**Fig. 2:**
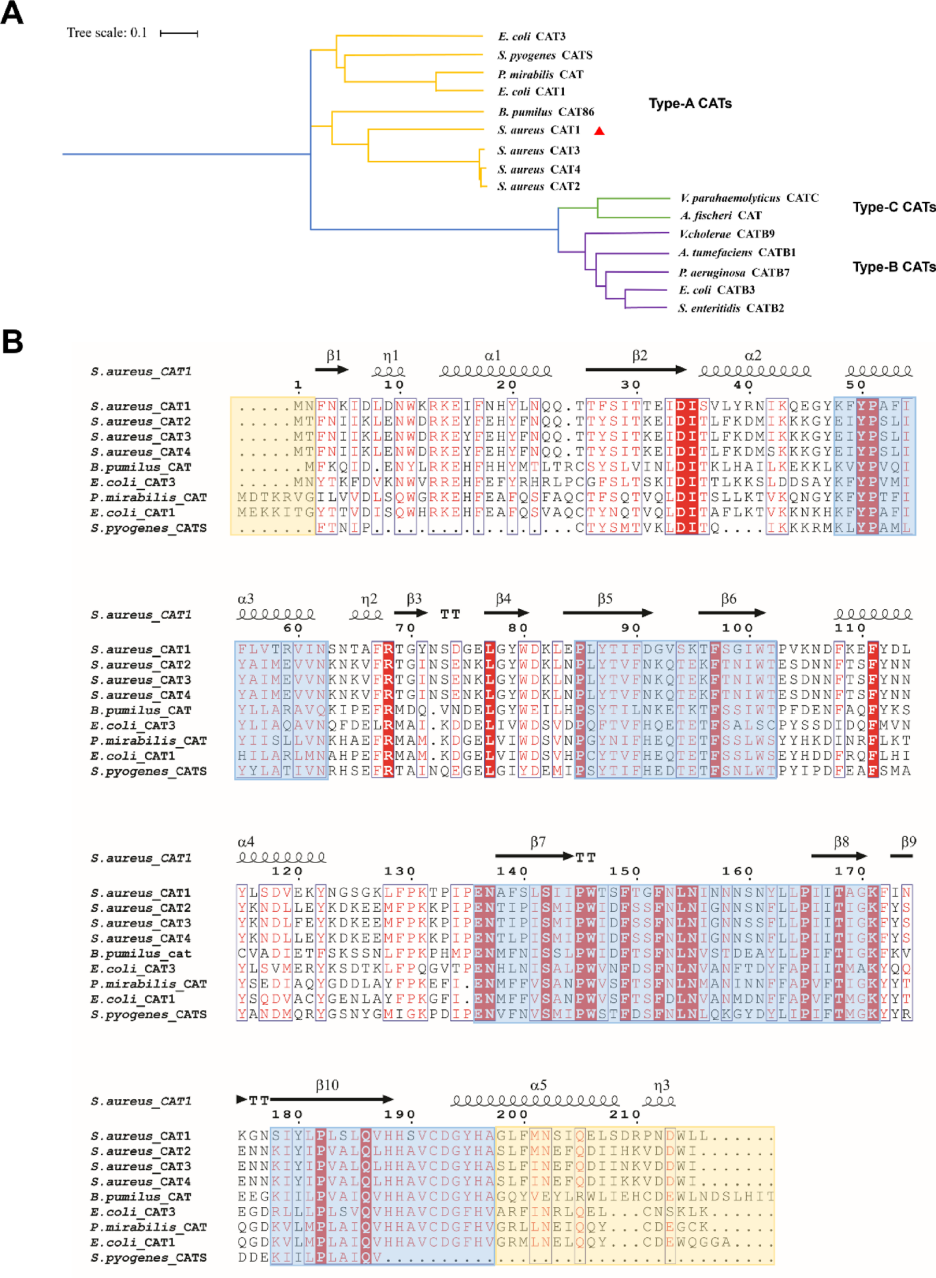
Phylogenetic tree of type-A, -B and -C CATs and sequence alignment of type-A CATs. (**A**) Yellow nodes represent type-A CATs, purple nodes represent type-B CATs, and green nodes represent type-C CATs. The saCAT1 is indicated by a red triangle. (**B**) Multiple sequence alignment of saCAT1 with type-A CATs from various species. The secondary structure elements are assigned according to the saCAT1 structure determined in this study. Blue boxes highlight core regions of high conservation, while yellow boxes indicate variable regions primarily located at the N- and C-terminal. Protein sequences used in the alignment are listed in Supplementary Table 1.

To investigate the evolutionary relationship of saCAT1, we performed a multiple sequence alignment of saCAT1 and previously reported type-A, type-B, and type-C CATs, followed by the construction of a phylogenetic tree (Fig. 2A). In addition to saCAT1, the alignment included *E. coli* CAT1 (ecCAT1), *E. coli* CAT3 (ecCAT3), saCAT2, saCAT3, saCAT4, *Proteus mirabilis* CAT, *Bacillus pumilus* CAT86, *Streptococcus pyogenes* CATS, *Agrobacterium tumefaciens* CATB1, *Salmonella enteritidis* CATB2, *E. coli* CATB3, *Pseudomonas aeruginosa* CATB7, *Vibrio cholerae* CATB9, *Aliivibrio fischeri* CAT, *Vibrio parahaemolyticus* CATC (Supplementary Table 1).

The results indicate that the four saCAT enzymes (saCAT1, saCAT2, saCAT3, and saCAT4) exhibit a high degree of phylogenetic relatedness, clustering collectively within the type-A CAT clade (Fig. 2A, yellow branch), which implies significant structural and functional conservation across these enzymes. In contrast, saCAT1 shows a distinct evolutionary divergence compared to type-B and type-C CATs, potentially indicative of species-specific adaptations or functional innovations. Notably, the fact that the four CATs from *S. aureus* form a single monophyletic group underscores their shared evolutionary ancestry. Pairwise sequence alignment revealed that saCAT1 shares 53–54% amino acid sequence identity with the other three saCATs, reflecting substantial sequence divergence. However, the high positive match rate (~ 73%) suggests that the physicochemical properties of most residues are conserved. This pattern strongly suggests the preservation of critical structural motifs and catalytic residues among these enzymes, which may underpin their enzymatic activity and functional roles.

Further sequence alignment of saCAT1 with other type-A CAT enzymes revealed significant conservation within the substrate-binding pocket regions previously reported, specifically spanning residues 48–62, 85–102, 136–171, and 178–197 (Fig. 2B, blue boxes). These regions are well aligned with high sequence identities. The preservation of shared structural framework accords with prior findings that the key catalytic residues responsible for enzymatic activity in type-A CATs are embedded within these conserved domains^15,21^. In contrast, sequence variability was primarily observed in the N- and C-terminal regions (Fig. 2B, yellow boxes), probably reflecting evolutionary divergence among type-A CAT enzymes. Collectively, these observations confirm that saCAT1 is a canonical member of the type-A CAT family, retaining conserved structural and functional features while exhibiting distinct sequence characteristics.

### Crystal structure of saCAT1

**Fig. 3:**
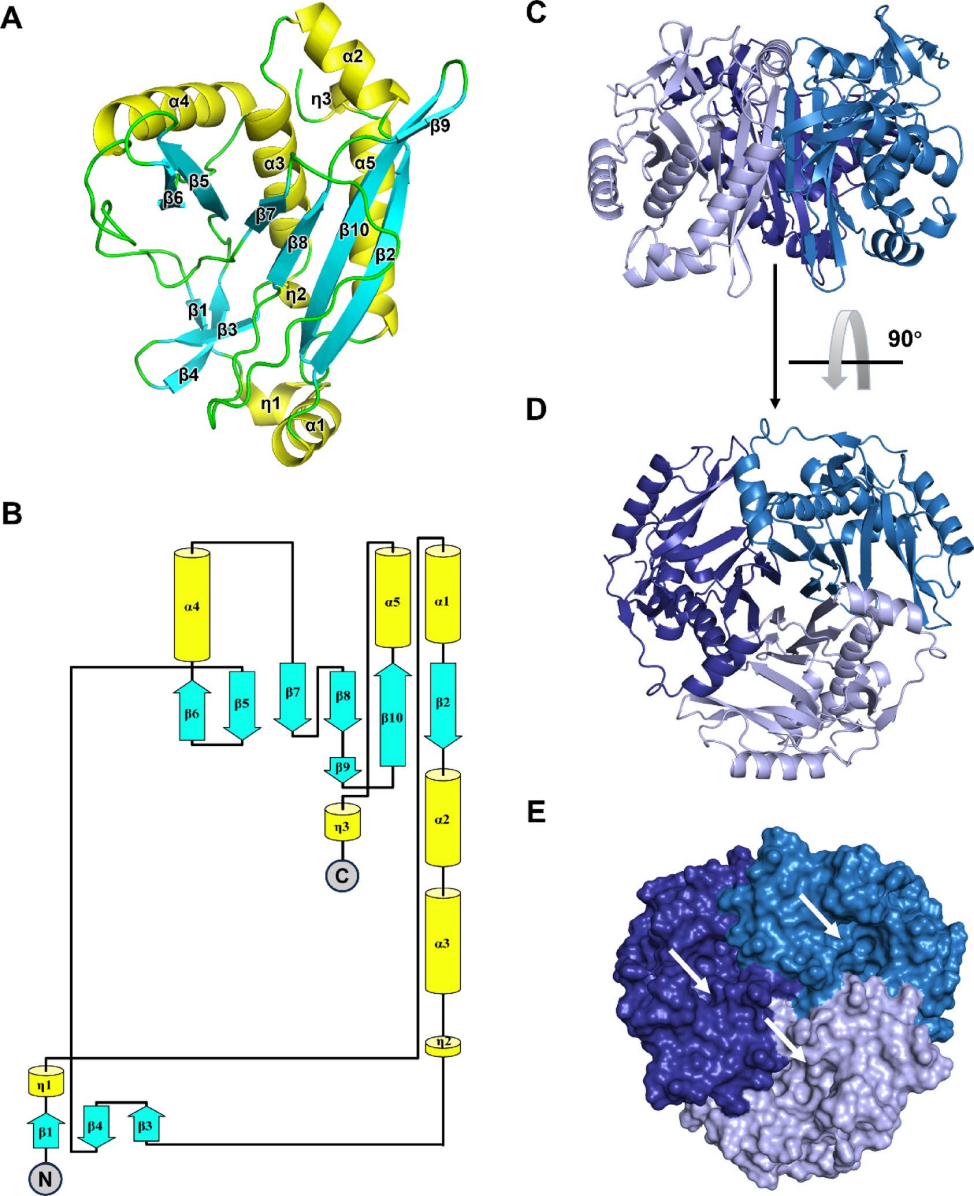
Crystal structure of saCAT1. (**A**) Tertiary structure of saCAT1 in ribbon diagram, with secondary structural elements colored in yellow for helixes and cyan for β-strands and labeled as in Fig. 1B. (**B**) Topology diagram of saCAT1, colored as in panel A. (**C**) Side view of a saCAT1 homotrimer, where the three protomers are colored in light purple, dark blue, and dark purple, respectively. (**D**) Top view of a saCAT1 homotrimer. (**E**) Surface representation of a saCAT1 homotrimer at top view, deep cavities indicated by white arrows.

To structurally characterize saCAT1, we determined its atomic-resolution structure by the means of x-ray crystallography. The crystals of saCAT1 belong to the space group *P*321 (Supplementary Fig. 1). The phasing problem was solved by molecular replacement with the structure of ecCAT3 (PDB entry: 3CLA^21^ as the search model. The final model of saCAT1 was refined to a resolution of 1.8 Å (*R*_work_/*R*_free_ = 0.150/0.166, Table 1), yielding a well-defined electron density map that enabled unambiguous modeling of all residues (1-216). Like other type-A CATs, three saCAT1 molecules assemble a biological homotrimer, where its 3-fold symmetric axis coincides with the crystallographic 3-fold rotational axis. Consequently, there is only one saCAT1 molecule in one asymmetric unit of the crystal. The general structure of saCAT1 adopts a canonical α/β fold with a three-domain architecture (Fig. 3A): (i) an N-terminal β-strand-rich domain primarily responsible for substrate binding; (ii) a central catalytic domain featuring a mixed α/β structure––centered on a twisted parallel β-sheet flanked by α-helices––that forms a compact catalytic core; and (iii) a predominantly α-helical C-terminal domain that stabilizes the overall structural stability and mediates inter-protomer interactions. The topology (Fig. 3B) and general structure of saCAT1 clearly exhibit a type-A CAT’s folding architecture, with a high structural similarities with previously reported type-A CAT (r.m.s.d. of 0.82 Å and 0.65 Å with ecCAT1^15^ and ecCAT3^21^, respectively).

**Table 1:** Data collection and refinement statistics.

	saCAT1
Data collection	
Space group	P 3 2 1
Cell constants	
Wavelength (Å)	1.5418
a, b, c (Å)	103.6 103.6 46.3
α, β, γ (°)	90 90 120
Resolution (Å)	32.2–1.8
Data completeness (%)	97.2 (77.2)
Redundancy	6.9
*R*_merge_	0.04
I/σ	15.4 (3.9)
Number of unique reflections	25,985
Refinement	
Resolution (Å)	32.2–1.8
*R*_work_/*R*_free_	0.150/0.166
*R*_free_ test set (%)	4.92
Wilson *B*-factor (Å^2^)	18.3
Bond length r.m.s.d. from ideal (Å)	0.007
Bond angle r.m.s.d. from ideal (°)	0.967
Total number of atoms	3941
Average *B*-factor of all atoms (Å^2^)	25.5

The values in parentheses represent the statistics for the highest resolution shell.

During the purification of saCAT1, gel filtration chromatography indicated that the protein exists as homotrimers in solution (Supplementary Fig. 2). Consistently, structural interface analysis via PISA^26^ (Proteins, Interfaces, Structures and Assemblies) further confirmed that the only biologically relevant oligomeric state of the saCAT1 molecules in the crystal is a trimeric state (Fig. 3C and D). This biological assembly exhibits exceptional thermodynamic stability, evidenced by strong inter-protomer interactions (ΔG_int_ = − 78.9 kcal/mol, calculated using PISA), deep cavities can be readily observed at the inter-protomer interfaces (Fig. 3E, indicated by white arrows), a substantial buried solvent-accessible surface area (SASA) at the interface (5,975.7 Å², three interfaces in total), and a high dissociation energy (ΔG_diss_ = 39.7 kcal/mol).

To define the structural basis of saCAT1 homotrimerization, we conducted a detailed interface analysis using PISA. In the final model of saCAT1 trimer, the analysis identified a major inter-protomer interface stabilized by 10 hydrogen-bonds, involving the following key residue pairs: A:Lys95/B: Tyr195, A: Leu128/B: Glu15, A: Thr150/B: Thr30(two hydrogen-bonds), A:Asn153/B: Ser28, A: Asn153/B: Asn153, A: Asn155/B: Thr26, A: Asn155/B: Asn153, A: Asn157/B: Gln24, and A: Asn158/B: Gln23 (where A and B denote distinct protomers). This interface buries approximately 1,991.9 Å² of SASA, accounting for 17.9% of the total SASA of two interacting saCAT1 protomers (Supplementary Table 2). The considerable buried area demonstrates a stable inter-protomer contact for trimer integrity.

Beyond the overall structural similarity, saCAT1 assembles into homotrimers through an identical architectural arrangement as previously reported type-A CAT structures (Supplementary Fig. 3). Comparative analysis of the trimeric interfaces revealed both conserved features and notable differences among saCAT1, ecCAT3, and ecCAT1. The ecCAT3 inter-protomer interface is stabilized by 19 hydrogen bonds and 2 salt bridges and buries 2,158.4 Å² of SASA (19.9% of total SASA). The ecCAT1 inter-protomer interface involves 19 hydrogen bonds and 3 salt bridges and buries a significantly larger 2,178.3 Å² of SASA (19.2% of total SASA). In comparison, the saCAT1 interface possesses fewer stabilizing interactions and a reduced buried area (1991.9 Å², 17.9%) relative to ecCAT3 and ecCAT1, suggesting lower interfacial stability. Nevertheless, the shared homotrimeric topology and core interfacial geometry underscore a high degree of structural conservation within the type-A CAT family, supporting a conserved functional assembly (Supplementary Table 2).

### Structural analysis of the chloramphenicol-binding site

Extesive evidences suggest that type-A CATs are functionally dependent on their homotrimeric configuration, wherein the trimeric conformation is essential for catalytic activity. Multiple structural studies have demonstrated that the catalytic active sites are formed directly at the inter-protomer interfaces; key elements contributed by adjacent protomers collectively create each complete active site^15,19,22,24^. Notably, the structural evidence available to date suggests that the substrate (chloramphenicol) binding induces no significant conformational changes in type-A CATs^15,21,24^. Given this rigidity, the saCAT1 holo structure determined in this study enables robust investigation of chloramphenicol binding through computational approaches, circumventing current limitations in obtaining substrate-bound saCAT1 crystals.

**Fig. 4:**
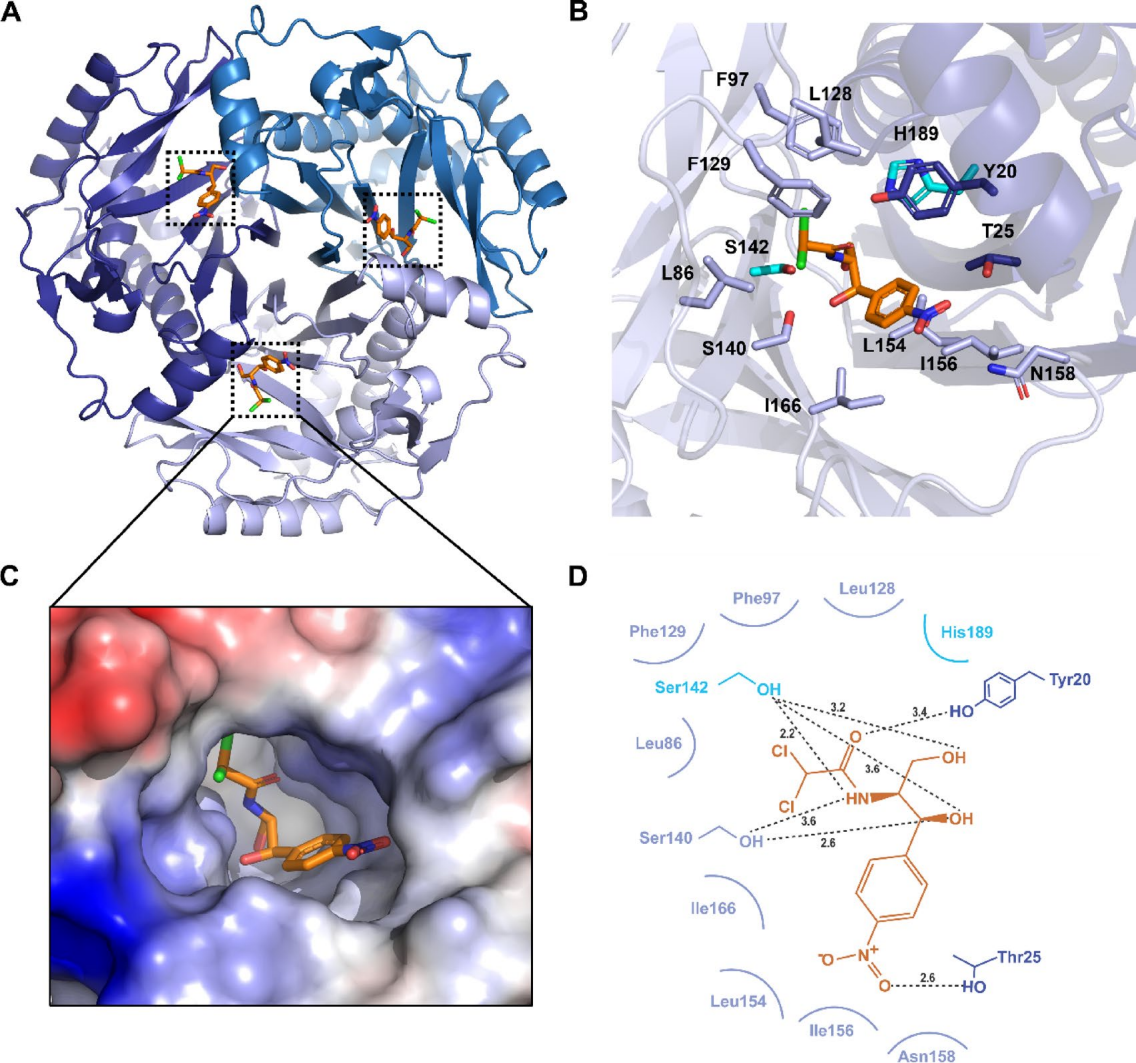
Structural analysis of the chloramphenicol-binding site. (**A**) There are three chloramphenicol-binding sites (indicated with dotted black boxes) located at the inter-protomer interfaces of a saCAT1 homotrimer. The chloramphenicol molecule is shown as stick diagram, with carbon in orange, nitrogen in blue, oxygen in red, and chlorine in green. (**B**) A zoom-in view of saCAT1’s chloramphenicol-binding site, where residues involved in substrate binding are represented as sticks and colored as their own protomers (binding monomer and catalytic monomer are colored in light purple and dark purple, respectively). Highly conserved residues Ser142 and His189 are highlighted via cyan color. (**C**) Electrostatic surface potential representation of the chloramphenicol-binding pocket, where the bound substrate are represented as in panel A. (**D**) Schematic diagram of the interactions between the binding pocket residues of saCAT1 and the bound chloramphenicol. Hydrogen bonds and hydrophobic interactions are indicated by black dashed lines and curved lines, respectively.

To structurally characterize chloramphenicol binding within the pre-formed active site of saCAT1, we performed structural modeling and molecular docking based on the holo structure of saCAT1 trimers. Structural alignment with the ecCAT3:chloramphenicol complex (PDB entry : 3CLA; r.m.s.d. = 0.65 Å for 498 Cα atoms) confirmed high conservation of the catalytic pocket geometry and positioned chloramphenicol within the deep interfacial cavity of saCAT1 trimer, spatially coincident with the substrate location in ecCAT3 (Fig. 4A). This result demonstrates three chemically equivalent binding sites per trimer, consistent with the enzyme’s known stoichiometry of substrate recognition.

The subsequent molecular docking of chloramphenicol to the inter-protomer binding site of saCAT1 (AutoDock Vina) exhibits detailed binding mode of the substrate. The binding pocket is primarily composed of β-strands 5–8 from one protomer (referred to as binding protomer) and the α1 helix and β2 strand from the adjacent protomer (referred to as catalytic protomer) (Fig. 4B). Furthermore, the sequence region corresponding to the binding pocket exhibits a high degree of conservation compared to the chloramphenicol-binding pocket-related sequences reported in the ecCAT1 and ecCAT3 structures15 (Fig. 2B, blue boxes), indicating the conservation of the chloramphenicol-binding mode. Electrostatic potential mapping reveals charge complementarity critical for substrate orientation: (i) the central hydrophobic zone accommodates the dichloroacetyl group of bound chloramphenicol; and (ii) positively charged residues around the binding pocket engage chloramphenicol’s nitro moiety, and anionic peripheries coordinate hydroxyl groups. Overall, the saCAT1 binding pocket provides an electrostatic environment conductive to the stable association of chloramphenicol (Fig. 4C).

Detailed structural mapping of the chloramphenicol binding site identified 13 residues directly involved in the substrate binding (Fig. 4B). The binding protomer contributes seven hydrophobic residues (Leu86, Phe97, Leu128, Phe129, Leu154, Ile156, Ile166) and three polar residues (Ser140, Ser142, Asn158), seven hydrophobic residues form a solvent-shielded cavity that enhances binding affinity through minimizing solvent exposure and stabilizing the bound chloramphenicol. This hydrophobic core region is further supplemented by the three polar residues. The partner catalytic protomer provides three key residues: Tyr20 and Thr25, which participate in hydrogen bonding, and the essential catalytic base His189. Collectively, Ser140 and Ser142 (from binding protomer) along with Tyr20 and Thr25 (from catalytic protomer) form seven hydrogen bonds with chloramphenicol’s polar groups, critically optimizing the orientation of chloramphenicol and anchoring it within the binding pocket (Fig. 4D). Notably, Ser142 is a highly conserved residue, spatially aligned with the corresponding catalytic serines at the active centers of ecCAT1 and ecCAT3 (Ser146 and Ser148, respectively)^15,21^. The critically positioned His189 (equivalent to His193 in ecCAT1 and His195 in ecCAT3) is known to be a highly conserved catalytic residue (Fig. 2B)^27,28^. Functioning as a general base catalyst, it activates chloramphenicol’s hydroxyl group to initiate the acetyl transfer reaction. Beyond its catalytic role, this residue further stablizes the transition state and maintains the precise geometry of the active site, rendering its indispensable role across type-A CAT enzymes^15,21,29,30^.

### Structural analysis of the FA-binding site

**Fig. 5:**
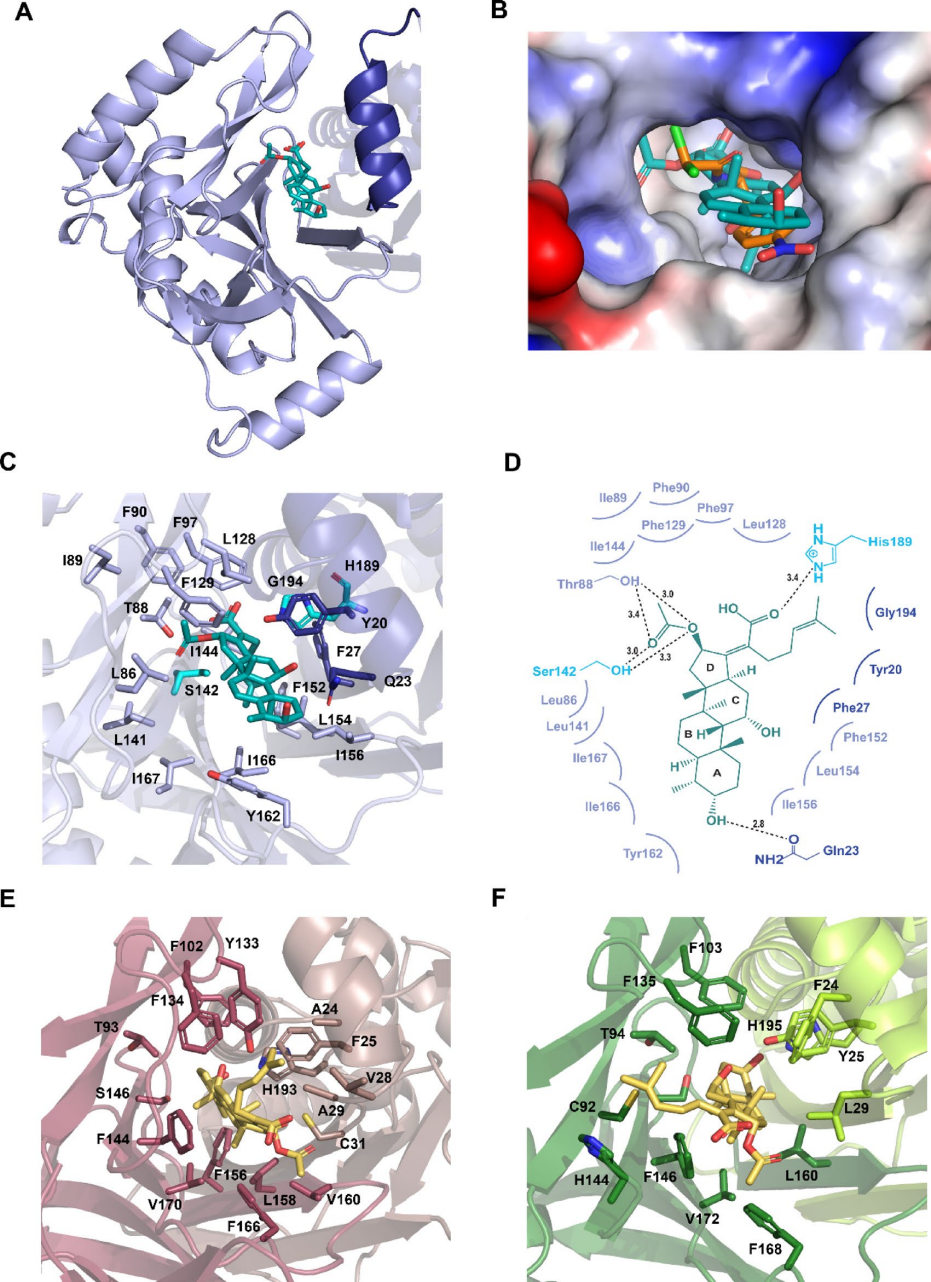
Structural analysis of the FA-binding site. (**A**) An FA molecule (cyan) docked to the inter-protomer pocket of a saCAT1 homotrimer. (**B**) Electrostatic surface potential representation of the FA-binding pocket of saCAT1, with superimposed FA (cyan) and chloramphenicol (orange) shown as sticks. (**C**) A zoom-in view of saCAT1’s FA-binding site, where residues involved in substrate binding are represented as sticks and colored as their own protomers. (**D**) Schematic diagram of the interactions between the binding pocket residues of saCAT1 and the bound FA. Hydrogen bonds and hydrophobic interactions are indicated by black dashed lines and curved lines, respectively. (**E**) A zoom-in view of ecCAT1’s FA-binding site. (**F**) A zoom-in view of the FA binding site of ecCAT3 quadruple mutant.

According to previous reports, certain type-A CATs possess unique structural properties that enable them to mediate the acetylation of larger steroid-like antibiotics, such as FA (Fig. 1A)^15,20,31,32^. To investigate the potential binding capability of saCAT1 toward FA, we performed molecular docking of FA into the trimeric structure of saCAT1 (AutoDock Vina). The results indicated that FA can stably occupy the chloramphenicol-binding pocket at the inter-protomer interface of saCAT1 homotrimer, exhibiting favorable binding affinity (ΔG = -6.321 kcal/mol) and specific interaction features (Fig. 5A). Notably, spatial coincidence of FA and chloramphenicol binding sites (Fig. 5B) suggests that FA could act as a competitive inhibitor by sterically obstructing chloramphenicol access^33^, potentially explaining the cross-resistance phenotypes in *S. aureus*.

Detailed structural analysis of the FA-binding site reveals a binding pattern similar to chloramphenicol-binding (Fig. 5C). The hydrophobic steroid ring system of FA makes numerous hydrophobic contacts with 14 residues from the binding protomer (Leu86, Ile89, Phe90, Phe97, Leu128, Phe129, Leu141, Ile144, Phe152, Leu154, Ile156, Tyr162, Ile166 and Ile167) and three residues from the catalytic protomer (Tyr20, Phe27 and Gly194). This hydrophobic core creates a suitable microenvironment to enhance the binding entropy through desolvation. Around the hydrophobic enclosure, four residues—Gln23, Thr88, Ser142, and His189—establishes precise polar anchoring for the bound FA. Critically, the δ-carbonyl oxygen of Gln23 forms a strong hydrogen bond (2.8 Å) with the hydroxyl group on the ring A of FA, effectively holding the head region of the substrate. Thr88 and Ser142 form hydrogen bonds with the acetoxy group of FA, while His189 interacts with the carboxyl group at the tail of FA, further stabilizing the substrate within the pocket (Fig. 5D).

Further structural comparison of FA: saCAT1 model with previously reported FA-bound type-A CAT complex structures (FA: ecCAT1 and FA: ecCAT3 quadruple mutnat) unveils an intriguing inversion of the orientaion of the bound FA. Despite overall structural similarity, saCAT1, ecCAT3 and the quadruple mutant of ecCAT3 (Q92C/N146F/Y168F/I172V) harbor FA in the same binding pocket but with dramatically different binding patterns. In FA: saCAT1 complex, the carboxylic acid group on the bound FA ring D and its 2-methylhex-2-ene tail are deeply buried within a hydrophobic cleft formed by residues Phe90, Phe97, Ile144, Phe152, and Leu154, retaining a relatively low solvent exposure of the substrate. By contrast, both FA: ecCAT1 and FA: ecCAT3 quadruple mutant complexes exhibit complete solvent exposure of this moiety (Fig. 5E and F). In ecCAT1, residue Phe134 from the binding protomer and Ala24 and Val28 from the catalytic protomer form a hydrophobic region near the entrance of the FA-binding pocket, which encloses the “tail” of FA and determines its conformation. Notably, wild-type ecCAT3 is unable to bind FA^33^. However, substitution of four residues with the corresponding residues from ecCAT1 appears to relax the structural rigidity of the wild-type ecCAT3, introducing a local distortion in the protein backbone. This alteration creates hydrophobic volume accommodating FA’s 2-methylhex-2-ene tail, allowing FA to occupy the reconfigured pocket (Figs. 5F). In the structure of the ecCAT3 quadruple mutant, Tyr25 forms a hydrogen bond with the hydroxyl group on ring A of FA, stabilizing the orientation of the ligand and ultimately enabling FA binding by the ecCAT3 mutant^15,33^. At the same position, the corresponding residue in saCAT1 is Gln23, which is also involved in forming hydrogen-bonds with the bound FA (Fig. 5D), exhibiting highly similar stabilization strategies. These findings provide structural insights into the differential substrate selectivity of type-A CATs toward FA.

### Enzymatic activity of saCAT and inhibition by FA

**Fig. 6:**
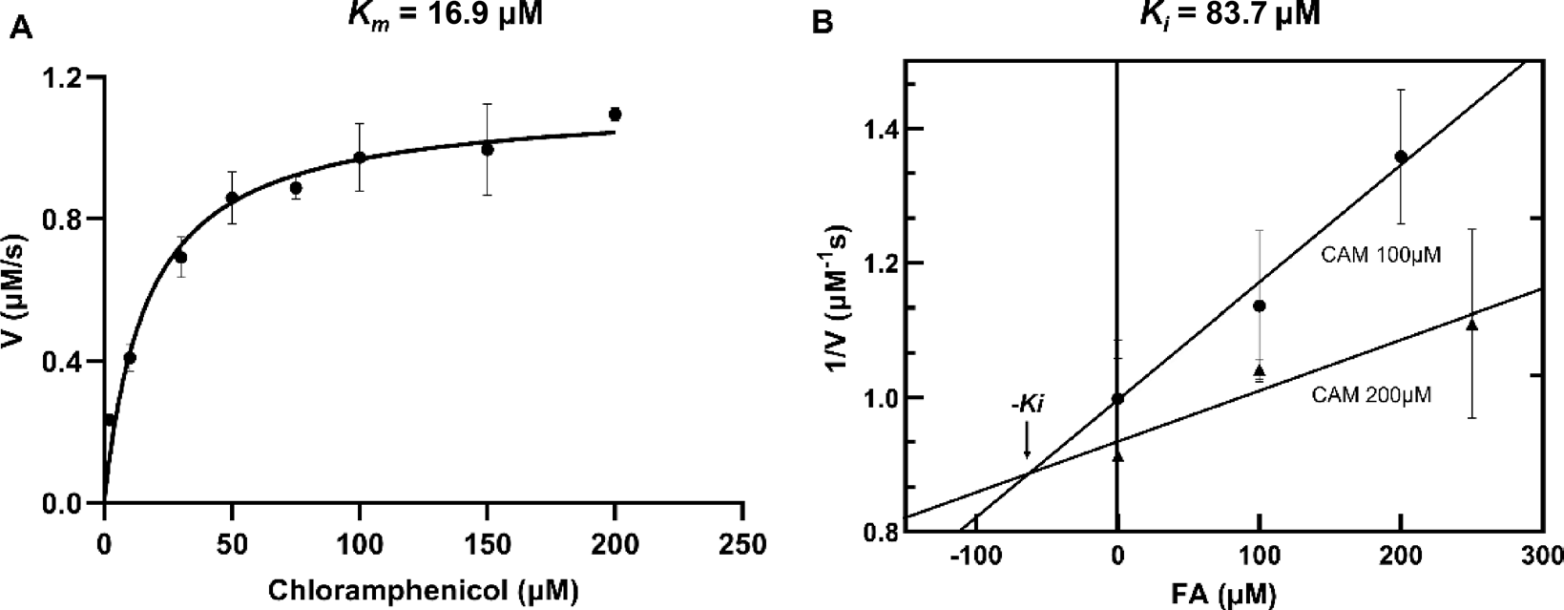
Enzyme kinetics and inhibition analysis of saCAT. (**A**) Michaelis–Menten curve of CAT-catalyzed acetylation of chloramphenicol. The reaction velocities were measured at various chloramphenicol concentrations, and the kinetic parameters were obtained by nonlinear regression fitting. Data points represent the mean values from three independent experiments. The Michaelis–Menten constant was estimated as Km = 16.9 ± 3.0 µM. (**B**) Enzyme activity in the presence of different concentrations of FA, showing its inhibitory effect. The initial velocities were measured after 2 min of reaction using a DTNB-based colorimetric assay at 412 nm. Data points represent the mean values from three independent experiments.Data were fitted by linear regression using GraphPad Prism 10.4.1. The slopes were statistically significant at both concentrations (CAM 100 µM: *P* < 0.05, R^2^ = 0.7532; CAM 200 µM: *P* < 0.05, R^2^ = 0.5271).

To comprehensively evaluate the catalytic properties of saCAT, we employed a colorimetric assay based on 5,5′-dithiobis (2-nitrobenzoic acid) (DTNB). In the presence of acetyl-CoA, saCAT catalyzes the acetylation of chloramphenicol, generating Coenzyme A (CoA) as a reaction product. CoA contains a free sulfhydryl group, which reacts with DTNB to produce a yellow-colored product measurable at 412 nm. Thus, the rate of absorbance increase corresponds to the rate of free sulfhydryl release, providing a direct quantification of product formation and thereby enzymatic activity. Chloramphenicol was used as the acetyl group acceptor substrate for enzymatic kinetic analysis. Initial reaction velocities were determined across a series of chloramphenicol concentrations, and the data were fitted to the Michaelis-Menten model using nonlinear regression analysis (Supplementary Fig. 4). The apparent Michaelis constant (*K*_m_) for saCAT was calculated to be 16.9 ± 3.0 µM (Fig. 6A), indicating a moderate substrate affinity. This result falls within the previously reported *K*_*m*_ range of bacterial chloramphenicol acetyltransferase enzymes for chloramphenicol (typically 5–50 µM), supporting the functional integrity of the recombinant saCAT used in this study^10,14^.

Previous studies have identified FA as a potential inhibitor of type-A CAT enzymes^15^. To assess the inhibitory effect of FA on saCAT, inhibitory assays were performed in the presence of varying concentrations of FA, and the corresponding reaction rates were measured. The inhibition constant (*K*_*i*_) of FA was determined to be 83.7 µM, which is significantly higher than the *K*_*m*_ value of 16.9 µM, indicating that FA exhibits a relatively weak inhibitory effect under the experimental conditions (Fig. 6B). Although saCAT does not catalyze FA, the elevated *K*_i_ value indicates that FA interacts with saCAT to some extent. The inhibitory mechanism of FA may involve partial occupation of the chloramphenicol-binding site, thereby indirectly interfering with substrate binding or inducing conformational changes in the enzyme, ultimately affecting catalytic efficiency. Given that the inhibitory effect of FA on saCAT is relatively weak, it highlights the need for further efforts to develop and optimize more effective alternative inhibitors in the future.

## Discussion

Chloramphenicol is a potent broad-spectrum antibiotic historically employed against diverse microbial infections. Yet, due to severe adverse effects, it was classified as a “possible human carcinogen” by the World Health Organization in 2007^34^. Amidst the escalating antimicrobial resistance crisis, there has been a renewed interest in reevaluating previously discontinued antibiotics, including chloramphenicol^35^. To date, two bacterial resistance mechanisms against chloramphenicol have been identified: (1) enzymatic reduction of its nitrobenzyl moiety to an amino group by nitroreductases^36,37^, and (2) acetylation of its 3-hydroxyl group by CATs, with acetyl-CoA as an acetyl donor^15,19^. Enzymatic inactivation of chloramphenicol and consequent steric hindrance of ribosomal binding is considered the predominant molecular mechanism of resistance in clinically significant pathogens^38^.

CATs are currently classified into three types (A, B, and C) based on structural and sequence similarities. Sequence analysis and phylogenetic comparison unequivocally establish saCAT1 as a canonical type-A CAT. While its close phylogenetic clustering with other saCATs (saCAT2-4) and their moderate sequence identity (53–54%) indicate a shared evolutionary origin, divergence in specific regions likely reflects adaptation to strain-specific functional demands. Crucially, high conservation within core secondary structural elements supports the idea that the structural scaffold critical for enzymatic function is evolutionarily conserved among type-A CATs. Conversely, sequence variability observed at the N- and C-termini and loop regions suggests mechanisms for functional diversification, potentially modulating substrate accessibility, catalytic efficiency, or regulatory interactions. These variable regions could be associated with species-specific adaptations and reflect evolutionary fine-tuning of saCAT1 function. In summary, these findings confirm that saCAT1 maintains the conserved features of type-A CATs while exhibiting structural plasticity that may contribute to its specific role in *S. aureus* antibiotic resistance.

Both in solution and crystals, saCAT1 exists as stable homotrimers, confirmed by gel filtration chromatography and PISA interface analysis. Similar to other type-A CAT enzymes, the trimeric configuration is considered essential for saCAT1’s biological function^15,21^. The trimer interface of saCAT1 is stabilized by a hydrogen-bond network involving key residues such as Lys95, Asn153, and Thr150. The binding of substrate chloramphenicol occurs within a pocket formed between two adjacent protomers of a homotrimer. This structural arrangement implies that a single saCAT1 monomer is unlikely to form either a complete substrate-binding pocket or a properly organized catalytic center, thereby resulting in complete loss of enzymatic activity. Trimeric assembly not only stabilizes the overall conformation of the enzyme but also enhances chloramphenicol recognition and acetylation efficiency through cooperative interactions^27,39^. Moreover, mutagenesis studies of ecCAT3 demonstrate that disrupting inter-protomer contacts markedly impairs its catalytic activity, further underscoring the critical role of homotrimerization in catalysis^15,40–42^. Future studies targeting interface residues (e.g., Asn153, Lys95) could further elucidate the mechanistic role of homotrimerization in substrate acetylation and resistance development.

The chloramphenicol binding site of saCAT1 exhibits a highly conserved binding mode, where the bound substrate is stabilized through hydrogen bonding and hydrophobic interactions, confirming that saCAT1 employs a canonical type-A CAT catalytic mechanism. Notably, subtle conformational adjustments in key residues (e.g., His189 and Ser142) suggest microevolutionary adaptations in *S. aureus*. Interestingly, a potential FA binding pocket was identified within the structure of saCAT1 homotrimer via molecular docking. As a steroidal antibiotic, FA can occupy the same binding site as chloramphenicol and may function as a competitive inhibitor of chloramphenicol acetylation. Enzymatic inhibition assays confirmed that FA exhibits a moderate inhibitory effect on saCAT1, with an inhibition constant of 83.7 µM, indicating a relatively weak binding affinity and limited inhibitory efficacy. These findings highlight the need for structure-based design and development of novel, more potent inhibitors targeting saCAT1, in order to overcome the limitations of current drugs and improve strategies against antibiotic resistance.

In this study, we report the first crystal structure of saCAT1, representing the first three-dimensional structure of any saCAT members. This finding addresses a critical gap in the structural landscape of type-A CATs, particularly from major pathogenic bacteria, and provides an essential structural framework for understanding chloramphenicol resistance in *S. aureus*. While establishing preliminary structure-function relationships for saCAT1, certain limitations persist: the static crystal conformation may not fully reflect the enzyme’s dynamic behavior in vivo, and the biological relevance of the identified FA-binding pocket requires experimental verification.

In conclusion, the structural elucidation of saCAT1 delivers the first atomic-level model of CAT-mediated resistance in *S. aureus*. This structure provides critical insights for the rational design of CAT-targeted inhibitors and the development of novel antibiotic combination therapies. Future work should explore the potential atypical substrate spectrum of saCAT1 and its role in clinical antibiotic resistance, consequently offering new avenues for targeted interventions against multidrug-resistant *S. aureus*, as well as other similar pathogens.

## Materials and methods

### Cloning

The target gene studied in this work is the *cat1* gene from the *Staphylococcus aureus* plasmid pC194 (GenBank: V01277.1, UniProtKB/Swiss-Prot: P00485). The *cat1* gene was cloned into the pFs91G expression vector, which contains an N-terminal 6×His tag and a thrombin protease recognition site. Following thrombin cleavage, four amino acids (GSLE) remain at the N-terminus of the protein. The recombinant plasmid was transformed into *Escherichia coli* BL21 (DE3) cells for subsequent protein expression.

### Protein expression and purification

Transformed *E. coli* cells were cultured in LB medium supplemented with 50 µg/mL gentamicin and expressed at 16 °C for 16–18 h. After harvesting the cells by centrifugation at 8,000 rpm for 10 min, resuspend the cells in a buffer containing 20 mM Tris–HCl (pH 8.0) and 100 mM NaCl. The cells were lysed by sonication and the lysate was clarified by centrifugation at 15,000 rpm for 30 min at 4 °C to obtain the soluble protein fraction. The fusion proteins were initially purified using a Ni-NTA affinity column (QIAGEN) and eluted with a buffer containing 20 mM Tris–HCl (pH 8.0), 100 mM NaCl, and 250 mM imidazole. The eluted fractions were incubated with thrombin at a ratio of 1:100 (w/w) at 4 °C overnight to remove the N-terminal His-tag. The cleaved target proteins were concentrated and further purified using Hi-trap Q (GE Healthcare) ion exchange chromatography and Superdex 200 Increase (GE Healthcare) size-exclusion chromatography. The final protein product had a purity greater than 90% and was concentrated to 10 mg/mL using a centrifugal concentrator (EMD Millipore).

### Protein crystallization, data collection and structure determination

Protein crystallization was performed using the hanging-drop vapor diffusion method at 16 °C. Initial crystallization screening was conducted using a Mosquito crystallization robot with 96-well plates. Commercial crystallization screens, including Crystal Screen, PEG/Ion, and MCSG1–4, were used. Two drops (0.5 µL each) were set up per well, with protein solution and reservoir solution mixed at a 1:1 volume ratio. The final protein concentrations in the drops were adjusted to 5 mg/mL and 10 mg/mL, respectively. Crystals suitable for structure determination appeared within 5–7 days. The optimized crystallization condition for CAT protein was 0.01 M cobalt (II) chloride hexahydrate, 0.1 M sodium acetate trihydrate (pH 4.6), and 1 M 1,6-hexanediol. Crystals were cryoprotected by adding 15% glycerol (v/v) to the reservoir solution prior to flash-cooling in liquid nitrogen. X-ray diffraction data were collected using a FR-X CCD 944HG X-ray diffraction system with a Cu Kα radiation source (λ = 1.5418 Å). Data indexing, integration, and scaling were performed using the HKL2000 software suite (version 706, HKL Research Inc., https://www.hkl-xray.com). Structure refinement was carried out using Phenix (version 1.20.1).

### Sequence alignment and phylogenetic tree

Multiple sequence alignment was performed using MEGA12, and a preliminary phylogenetic tree was constructed based on the aligned sequences. The resulting phylogenetic tree was visually enhanced using iTOL (https://itol.embl.de/) and Microsoft PowerPoint. Sequence alignment was generated with ESpript (http://espript.ibcp.fr/ESPript/ESPript/), which also annotated the secondary structural elements corresponding to the crystal structure of *S. aureus* CAT1 (PDB ID: 9M5S). The same set of protein sequences was used for both the alignment and phylogenetic analysis. Detailed information on the protein sequences included in the analysis is provided in supplementary Table 1.

### Molecular docking

Molecular docking was performed using AutoDock Vina version 1.2.7 to predict the binding conformation and affinity between the ligand and the receptor protein. The receptor structure was retrieved from the Protein Data Bank (PDB ID: 9M5S) and processed by removing water molecules and heteroatoms, followed by conversion to PDBQT format using AutoDock Tools (ADT). The ligand, chloramphenicol was prepared similarly. All torsions in the ligand were left flexible to allow full conformational exploration. The docking grid was defined to encompass the presumed binding site, with the grid box center set at coordinates (x = − 12.448, y = − 6.358, z = 14.559) and dimensions of 20 Å × 20 Å × 20 Å along the X, Y, and Z axes. Exhaustiveness was set to 8, and a maximum of 9 binding modes were generated. The output binding modes were ranked based on predicted binding free energy (kcal/mol), and the top-ranked pose was selected for further analysis. Binding poses were visualized and analyzed using PyMOL and AutoDock Tools to inspect potential interactions between the ligand and receptor residues.

### Enzyme kinetics and Inhibition assay

The enzymatic activity of saCAT was assessed using a DTNB-based colorimetric assay kit (Beyotime Biotechnology, China), which detects the release of CoA-SH upon acetyl group transfer. Reactions were carried out in 96-well plates at 37 °C in a total volume of 200 µL containing 180 μL Ellmans Reagent Solution, 0.14 mM saCAT1 enzyme, 0.2 mM acetyl-CoA, and varying concentrations of chloramphenicol (2–200 µM). The enzyme was preincubated in the reaction buffer, and the reaction was initiated by the addition of chloramphenicol. The absorbance at 412 nm was measured after 2 min using a microplate reader. For inhibition assays, FA was added to the reaction system at concentrations ranging from 0 to 250 µM. All assays were performed in triplicate. Initial reaction velocities were calculated and fitted to the Michaelis-Menten equation for *K*_*m*_ determination, and the inhibition data were analyzed using GraphPad Prism version 10.4.1 to determine the inhibition constant (*K*_*i*_) by fitting to a linear regression model.

## Supplementary Information

Below is the link to the electronic supplementary material.

Supplementary Material 1

## Data Availability

The atomic coordinates and structure factors for the *S. aureus* CAT1 have been deposited in the Protein Data Bank with accession number 9M5S. The data supporting the findings of this study are available from the corresponding author upon reasonable request.
