# Supplementary material for: Structural basis for antibiotic resistance by chloramphenicol acetyltransferase type A in Staphylococcus aureus
Source: Sci Rep. 2025 Oct 23;15:37020. doi: 10.1038/s41598-025-18365-4 (PMC12549896; doi:10.1038/s41598-025-18365-4)
Supplement: Supplementary file 1 — Supplementary Material 1 [file 41598_2025_18365_MOESM1_ESM.docx]

**Supplementary data**

**
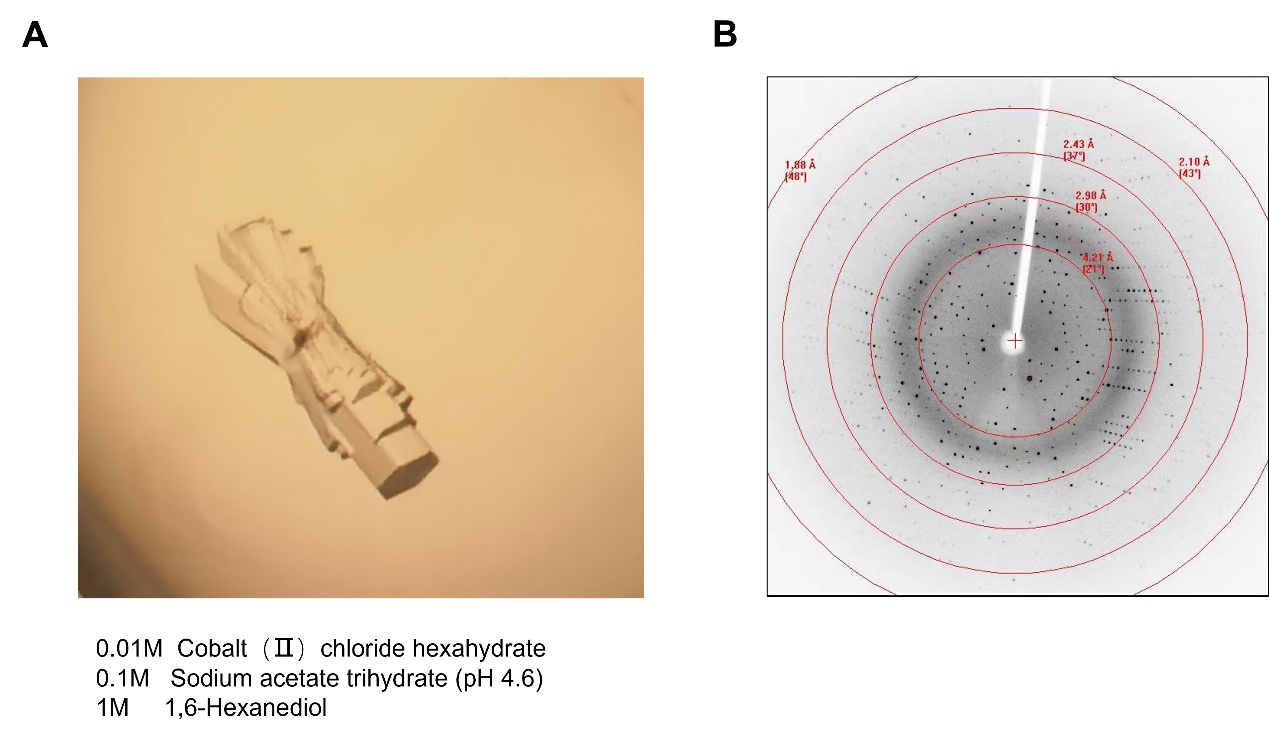
**

**Supplementary Figure 1 Selected saCAT1 crystal and corresponding X-ray diffraction pattern.**

A: Crystal of saCAT1 showing a hexagonal prism morphology. The crystal was obtained under the condition of 0.01 M cobalt (II) chloride hexahydrate, 0.1 M sodium acetate trihydrate (pH 4.6), and 1 M 1,6-hexanediol. B:X-ray diffraction pattern of the crystal collected using a RIGAKU FR-E X-ray source (λ = 1.5418 Å) and a RIGAKU SATURN 944+ detector.

**
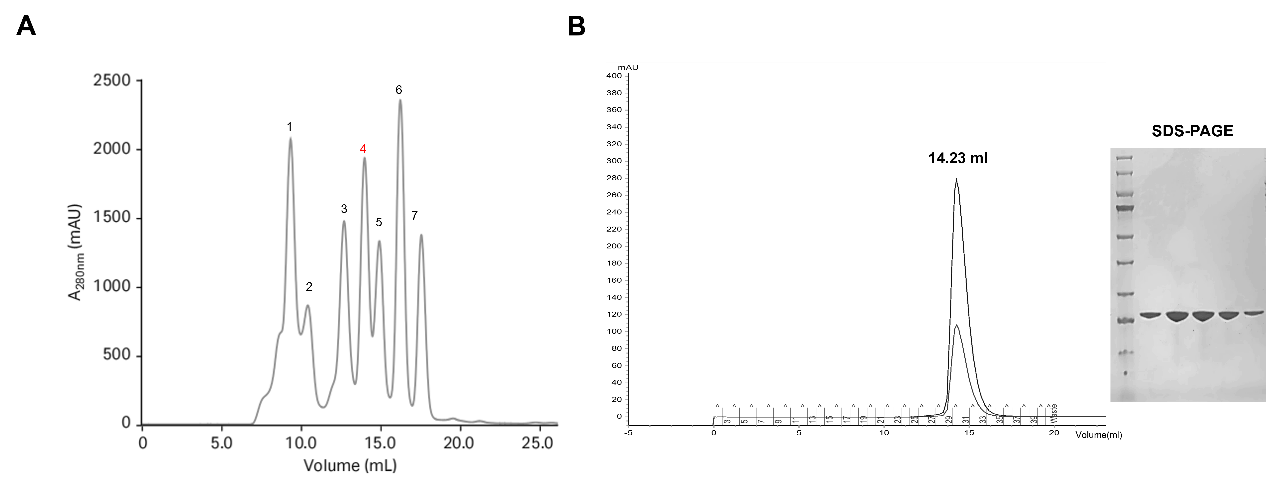
**

**Supplementary Figure 2 Size-exclusion chromatography profile of saCAT1.**

A:Superdex 200 Increase marker，Sample mix is 1. Thyroglobulin (Mr 669 000), 2. Ferritin (Mr 440 000), 3. Aldolase (Mr 158 000), 4. Conalbumin (Mr 75 000), 5. Ovalbumin (Mr 44 000), 6. Carbonic anhydrase (Mr 29 000), 7. Ribonuclease A (Mr 13 700), 3 mg/mL. B: *S. aureus* CAT1 was purified using a Superdex 200 Increase column. The monomeric molecular weight of saCAT1 is approximately 25 kDa, and the protein eluted at 14.23 mL, corresponding to an estimated molecular weight of ~75 kDa based on the Superdex 200 Increase calibration curve, indicating that CAT1 exists as a trimer in solution. SDS-PAGE analysis showed that the purity of the target protein was greater than 95%.

**
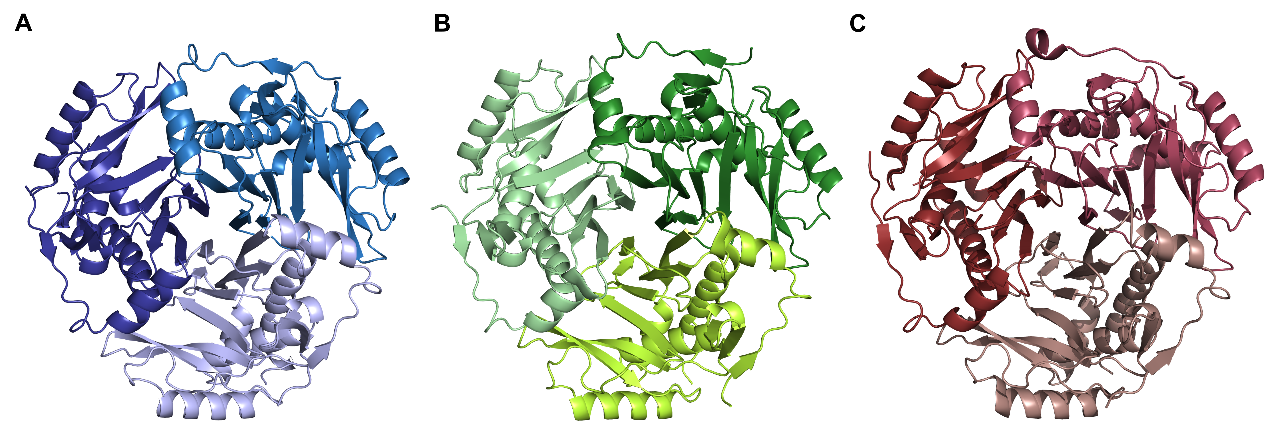
**

**Supplementary Figure 3 Conserved trimeric assembly of saCAT1 with ecCAT1 and ecCAT3.**

A: Top view of a saCAT1 homotrimer. B: Top view of a ecCAT3 homotrimer (PDB ID: 3CLA^21^). C: Top view of a ecCAT1 homotrimer (PDB ID: 3U9F^15^).


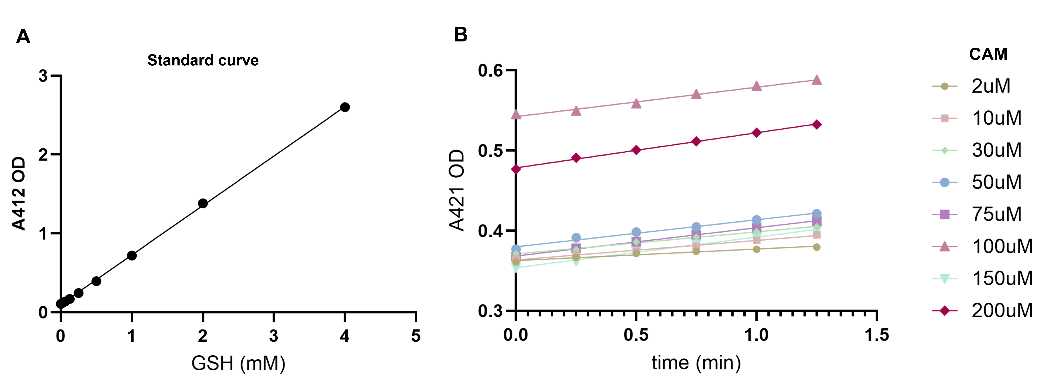


**Supplementary Figure 4 Standard curve for the DTNB-based colorimetric assay and reaction curves of saCAT.**A: A linear relationship was established between the absorbance at 412 nm and the concentration of free thiol (CoA-SH) released in the reaction. This standard curve was used to calculate the initial velocities in subsequent enzyme activity assays. B: Reaction curves of saCAT at different concentrations of chloramphenicol. The increase in absorbance at 412 nm over time reflects the rate of acetyl group transfer. Reaction rates at each chloramphenicol concentration were used to construct the Michaelis-Menten kinetic curve (shown in Figure 6A).

**Supplementary Table 1 Summary of representative Type-A, -B, and -C CATs**

| **Type** | **Protein Name** | **Bacterial source(s)** | **GenBank ID** | **PDB ID** | **References** |
| --- | --- | --- | --- | --- | --- |
| A-1 | CAT1 | *Escherichia coli* | V00622.1 | 1NOC/1PD5/1Q23/  3U9B/3U9F | ^43^ |
| A-2 | CAT3 | *Escherichia coli* | X07848.1 | 3CLA/6X7Q | ^16^ |
| A-3 | CAT2 | *Staphylococcus aureus* | X02529.1 |  | ^44,45^ |
| A-4 | CAT3 | *Staphylococcus aureus* | X02872.1 |  | ^46^ |
| A-5 | CAT4 | *Staphylococcus aureus* | X60827.1 |  | ^47^ |
| A-6 | CAT1 | *Staphylococcus aureus* | V01277.1 | 9M5S | ^48^ |
| A-7 | CAT | *Proteus mirabilis* | M11587.1 |  | ^49^ |
| A-8 | CAT86 | *Bacillus pumilus* | K00544.1 |  | ^50^ |
| A-9 | CATS | *Streptococcus pyogenes* | X74948.1 |  | ^51^ |
| B-1 | CATB1 | *Agrobacterium tumefaciens* | M58472.1 |  | ^17^ |
| B-2 | CATB2 | *Salmonella enteritidis* | AJ487034.1 |  | ^52^ |
| B-3 | CATB3 | *Escherichia coli* | AY259086.1 |  | ^53^ |
| B-4 | CATB7 | *Pseudomonas aeruginosa* | AF036933.1 | 1XAT/2XAT | ^54^ |
| B-5 | CATB9 | *Vibrio cholerae* | AF462019.1 | 3EEV/6PUA/  6PUB/6U9C | ^55^ |
| C-1 | CAT | *Aliivibrio fischeri* | AAW87860.1 | 5UX9/6PXA | ^56^ |
| C-2 | CATC | *Vibrio parahaemolyticus* | WP_025635165.1 |  | ^22^ |

**Supplementary Table 2 Comparison between three type-A CATs interfaces from *S. aureus* (left column), *E. coli* (middle column and right column). Different number of salt bridges, H-bonds and interface area can be seen between these enzymes.**

| **PDB ID:9M5S** | | | **PDB ID:3CLA** | | | **PDB ID:3U9F** | | |
| --- | --- | --- | --- | --- | --- | --- | --- | --- |
| **Number of H-bonds: 10** | | | **Number of H-bonds: 19** | | | **Number of H-bonds: 19** | | |
|  | | | **Number of Salt bonds: 2** | | | **Number of Salt bonds: 3** | | |
| **Buried SASA[Å^2^]: 5975.7** | | | **Buried SASA[Å^2^]: 6475.2** | | | **Buried SASA[Å^2^]: 6534.9** | | |
| **Proportion of total SASA：17.9%** | | | **Proportion of total SASA：19.9%** | | | **Proportion of total SASA：19.2%** | | |
| **Protomer 1** | **Dist.[Å]** | **Protomer 2** | **Protomer 1** | **Dist.[Å]** | **Protomer 2** | **Protomer 1** | **Dist.[Å]** | **Protomer 2** |
| A: LYS95[HZ1] | 1.83 | A: TYR195[OH] | A: LEU134[N] | 2.77 | A: GLU20[OE1] | H: ARG203[NH1] | 3.67 | G: GLU100[OE1] |
| A: LEU128[H] | 2.12 | A: GLU15[OE1] | A: ASP156[N] | 2.82 | A: THR36[O] | H: HIS21[NE2] | 2.73 | G: PHE102[O] |
| A: THR150[H] | 2.02 | A: THR30[O] | A: SER157[N] | 3.53 | A: THR36[O] | H: ASN206[ND2] | 3.49 | G: TRP150[O] |
| A: THR150[OG1] | 3.45 | A: THR30[O] | A: SER157[OG] | 3.10 | A: THR36[OG1] | H: THR154[OG1] | 2.74 | G: THR154[OG1] |
| A: ASN153[H] | 2.24 | A: SER28[O] | A: ASN159[N] | 2.93 | A: SER34[O] | H: THR36[N] | 3.27 | G: SER155[O] |
| A: ASN153[HD22] | 2.45 | A: ASN153[OD1] | A: ASN161[N] | 2.71 | A: GLY32[O] | H: SER155[OG] | 2.97 | G: SER155[OG] |
| A: ASN155[H] | 1.94 | A: THR26[O] | A: ASN161[ND2] | 3.22 | A: ASN159[OD1] | H: ASN34[N] | 3.16 | G: ASP157[O] |
| A: ASN155[HD21] | 2.31 | A: ASN153[OD1] | A: ALA163[N] | 3.07 | A: PRO30[O] | H: THR32[OG1] | 3.53 | G: ASN159[O] |
| A: ASN157[H] | 2.42 | A: GLN24[O] | A: GLU101[OE1] | 3.21 | A: ARG205[NH1] | H: THR32[N] | 2.88 | G: ASN159[O] |
| A: ASN158[HD22] | 2.07 | A: GLN23[OE1] | A: PHE103[O] | 2.85 | A: HIS21[NE2] | H: GLN35[OE1] | 3.41 | G: ASN148[ND2] |
|  |  |  | A: TRP152[O] | 3.07 | A: ASN208[ND2] | H: THR36[O] | 2.81 | G: THR154[N] |
|  |  |  | A: ASN154[O] | 2.77 | A: SER37[OG] | H: THR154[O] | 3.02 | G: THR154[OG1] |
|  |  |  | A: ASP156[OD2] | 3.42 | A: LYS38[NZ] | H: THR36[O] | 3.54 | G: SER155[N] |
|  |  |  | A: SER157[O] | 3.17 | A: THR36[N] | H: ASN34[O] | 3.00 | G: ASP157[N] |
|  |  |  | A: SER157[OG] | 3.50 | A: THR36[N] | H: THR32[O] | 2.91 | G: ASN159[N] |
|  |  |  | A: ASN159[O] | 2.91 | A: SER34[N] | H: THR32[OG1] | 3.20 | G: ASN159[ND2] |
|  |  |  | A: ASN161[O] | 2.82 | A: GLY32[N] | H: ASN159[OD1] | 3.48 | G: ASN159[ND2] |
|  |  |  | A: ASN161[OD1] | 3.20 | A: ASN159[ND2] | H: VAL160[O] | 3.88 | G: ASN159[ND2] |
|  |  |  | A: ASN161[OD1] | 3.38 | A: GLN192[NE2] | H: GLN30[O] | 2.95 | G: ALA161[N] |
|  |  |  | **Salt bridges** | | | **Salt bridges** | | |
|  |  |  | **Protomer 1** | **Dist.[Å]** | **Protomer 2** | **Protomer 1** | **Dist.[Å]** | **Protomer e2** |
|  |  |  | A: GLU101[OE1] | 3.21 | A: ARG205[NH1] | H: ARG203[NH1] | 3.67 | G: GLU100[OE1] |
|  |  |  | A: ASP156[OD2] | 3.42 | A: LYS38[NZ] | H: ARG203[NH2] | 3.80 | G: GLU100[OE1] |
|  |  |  |  |  |  | H: ARG203[NH1] | 3.93 | G: GLU100[OE2] |
